# Effect of Flavanol-Rich Cacao Extract on the Profile of Mood State in Healthy Middle-Aged Japanese Women: A Randomized, Double-Blind, Placebo-Controlled Pilot Study

**DOI:** 10.3390/nu15173843

**Published:** 2023-09-03

**Authors:** Rika Murakami, Midori Natsume, Kentaro Ito, Shukuko Ebihara, Masakazu Terauchi

**Affiliations:** 1 R&D Division, Meiji Co., Ltd., 1-29-1 Nanakuni, Hachioji, Tokyo 192-0919, Japan; midori.natsume@meiji.com (M.N.); kentarou.itou@meiji.com (K.I.); 2 Chiyoda Paramedical Care Clinic, 3-3-10 Nihonbashihongoku-cho, Chuo-ku, Tokyo 103-0021, Japan; s.e@cpcc.co.jp; 3 Department of Women’s Health, Tokyo Medical and Dental University, 1-5-45 Yushima, Bunkyo-ku, Tokyo 113-8510, Japan; teragyne@tmd.ac.jp

**Keywords:** middle-aged women, cacao flavanol, menopausal symptom, mood, vigor

## Abstract

To investigate the effects of flavanol-rich cacao extract on healthy middle-aged women’s fatigue and mood conditions, we conducted a randomized, double-blind, placebo-controlled study in women aged 40–60 years who had reported fatigue and had shown high levels of a serum oxidative stress marker. We randomized the participants (*n* = 60) into equal groups receiving either a beverage containing cacao flavanols (240 mg/200 mL/day) or a placebo for 8 weeks. Before and after the 8-week treatment, we determined the participants’ Chalder fatigue scale (CFS) scores, various mood states, autonomic nervous system (ANS) activity levels, and their ANS balance. The results demonstrated that among the mood states, the indicators of negative mood (e.g., depression, fatigue, and anger) and the total mood disturbance score were significantly lower in the cacao group compared to the placebo group after the treatment (*p* < 0.05). The change in the index of positive mood (i.e., vigor) from baseline to 8 weeks was significantly higher in the cacao group versus the placebo group (*p* < 0.05). There were no significant between-group differences in the changes in the CFS score or ANS activity level. The consumption of flavanol-rich cacao extract both suppressed negative moods and promoted positive moods in healthy middle-aged women. These results suggest that cacao flavanols may be a useful food material that can improve variable mood conditions in middle-aged women and support their active lives.

## 1. Introduction

Middle-aged women in the transition to menopause may experience symptoms such as fatigue, sleep disturbance, psychological problems, and vasomotor symptoms. In 2005, Satoh and Ohashi reported that nearly one-quarter of community-dwelling healthy Japanese women in the peri- and postmenopausal states suffered from menopausal symptoms that decreased their quality of life (QoL) [1]. Another study reported that 88.2% of the women who visited a menopause clinic complained of general fatigue as the most frequent symptom [2]. The relationship between fatigue and estrogen depletion and/or rapid estrogen fluctuations in middle-aged women is not yet fully understood and requires further study.

The flavanols derived from cacao beans are thought to have antioxidant properties and to contribute to cardiometabolic health by regulating blood pressure and lipid profiles [3]. In 2010, Scholey et al. showed that an acute administration of cacao flavanols significantly attenuated the mental fatigue caused by a high-load cognitive test battery [4]. A study of subjects with chronic fatigue syndrome also indicated that polyphenol-rich chocolate may improve symptoms [5]. Fatigue is well known to be associated with oxidative stress, and aging and menopause are both known to exacerbate oxidative stress [6,7]. Moreover, several psychological changes following menopause have been shown to exert a pro-oxidant effect [8].

We thus hypothesized that healthy middle-aged women with high levels of both oxidative stress and fatigue could achieve reductions in fatigue and mood changes by consuming cacao flavanols, which are antioxidants. We conducted this study to investigate the effect of flavanol-rich cacao extract on fatigue and mood in healthy middle-aged Japanese women.

## 2. Materials and Methods

### 2.1. Study Design

We conducted a randomized, double-blind, placebo-controlled, parallel-group trial from 19 March 2021 to 19 August 2021 at the Chiyoda Paramedical Care Clinic (Tokyo). The study protocol was approved by the Institutional Review Board of Chiyoda Paramedical Care Clinic (approval No. 21031902, Tokyo) and the Meiji Institutional Review Board (Tokyo) (approval No. 197) and was designed to comply with the Declaration of Helsinki. The protocol was also registered in the UMIN Clinical Trials Registry (UMIN000043966). Participants were recruited via the website of CPCC Co., Ltd. (Tokyo, Japan). Each woman provided written informed consent to participate before randomization. A data-monitoring committee assured us of the accuracy of data collection and inputs. All of the study procedures, including its explanation, written informed consent acquisition, questionnaire, and testing, were conducted in Japanese. The reporting of this trial follows the recommendations of the CONSORT (Consolidated Standards of Reporting Trials) 2010 statement [9].

### 2.2. The Participants and the Study Flow

As the results of the initial screening, a total of 108 Japanese healthy women were eligible to participate in the study when they met all of the following criteria: age 40–60 years, had been feeling fatigued in their everyday lives, had a body mass index (BMI) of 18.6–30 kg/m^2^, and had missed a menstrual cycle by ≥1 week or had ≤5 years of amenorrhea since the last menstrual period. Women who had food allergies, had difficulty eating cacao-containing foods, or were under treatment with an agent that may affect this study (e.g., estrogen, herbal medicine, psychotropic agents, or functional foods) were deemed ineligible for participation in the study. Sixty participants were selected based on their values on (i) the Chalder fatigue scale (CFS) and (ii) the derivatives of reactive oxygen metabolites (d-ROMs), obtained as described below. Specifically, we ranked all 108 of the potential participants from the highest to lowest total CFS score at baseline; the participants’ d-ROMs levels were similarly ranked from highest to lowest. The sum of the respective rankings was used as an individual’s overall rank, and the 60 participants were recruited from the highest-ranked group; if there was more than one participant with the same overall rank value at the 60th spot, the participant with the higher CFS score was selected.

The allocation manager (Okutoeru, LLC; Tokyo, Japan) stratified by CFS and d-ROMs values as allocation factors to randomize the participants to a cacao group and a placebo group in a 1:1 ratio (*n* = 30 each). The number of participants was based on similar studies [10,11]. The allocation table with each participant’s ID number and test-food code was prepared by the allocation manager and then sealed until the test-food representative unblinded the codes after the data were finalized at the case conference. Thus, both the participants and the investigators remained blinded throughout the study period in order to ensure double blindness.

The participants were then required to undergo three tests: a pre-intake test (Week 0), a midpoint test (Week 4), and a post-intake test (Week 8). In all three tests, the participants’ feeling of fatigue was evaluated at Weeks 0 and 8 only, the participants’ mood states and autonomic nervous system activity were measured.

### 2.3. Test Beverages

For 8 weeks (56  ±  2 days) from the day of the pre-intake test to the day before the post-intake test, each participant consumed either a beverage containing cacao flavanols or a placebo beverage that had a similar taste and appearance (200 mL per day). Both test beverages were manufactured by the R&D Division in Meiji Co., Ltd. (Tokyo, Japan). Flavanol-rich cacao extract was produced by treating cacao beans with water acidified with citric acid at 70 ± 20 °C for 5 h. The contents of each beverage were known only to the manufacturers, and the allocation was thus concealed from both the participants and the investigators. The cacao-flavanol content of the cacao beverage was analyzed by the Association of Official Agricultural Chemists (AOAC) method ‘2012.24 Flavanols and Procyanidins’ [12]. The cacao-flavanol content of the placebo beverage was <0 mg/200 mL, and that of the cacao beverage was 240 mg/200 mL. The cacao beverage contained 120 mg of catechin, epicatechin, procyanidin B2, procyanidin B5, procyanidin C1, and cinnamtannin A2 according to a high-performance liquid chromatography (HPLC) analysis of the ingredients [13]. Flavanol-rich cacao extract also contains methylxanthines. The cacao beverage contained 19 mg of caffeine and 106 mg of theobromine.

### 2.4. Measurements

#### 2.4.1. Body Composition and Blood Pressure

At the initial screening, each participant’s height, weight, and BMI were measured by a height and mass scale (A&D Medical, Tokyo, Japan), and blood pressure was measured using a TM-2656 VPW blood pressure monitor (A&D Medical, Tokyo, Japan).

#### 2.4.2. Serum Oxidative Stress Biomarkers

At the initial screening, the participants’ blood samples were collected and serum was obtained by centrifugation. The measurements of d-ROMs and the biological antioxidant potential (BAP) were obtained as described [14,15,16].

#### 2.4.3. Autonomic Nervous System Activity

At Weeks 0 and 8, the participants’ autonomic nervous system activity was measured with an acceleration plethysmogram (Artett C, U-Medica, Osaka, Japan). The participants maintained a sitting position with an arm at heart-level; they inserted the index or middle finger into the sensor body and the autonomic nervous system activity was then measured for 5 min. The low-frequency (LF) component, the high-frequency (HF) component, the ratio of these components (LF/HF), and the very-low-frequency (VLF) component were then determined by Artett CDN autonomic nervous system evaluation software (Ver.1.0.0.35, U-Medica, Osaka, Japan).

### 2.5. Questionnaire

#### 2.5.1. Measurement of Fatigue

At Weeks 0, 4, and 8, the participants’ feeling of fatigue was evaluated by the Chalder fatigue scale (CFS) [17], which is a self-administered questionnaire with 14 items that evaluate fatigue-related symptoms based on four possible responses: ‘better than usual’, ‘no more than usual’, ‘worse than usual’, and ‘much worse than usual’.

#### 2.5.2. Menopausal Symptoms

Before treatment, the participants’ menopausal symptoms were evaluated by the Menopausal Health-Related Quality of Life (MHR-QOL) Questionnaire, which was developed and validated at Tokyo Medical and Dental University Hospital and is a modification of the Women’s Health Questionnaire. The MHR-QOL contains 38 items scored on a four-point or binary scale covering four major domains of a women’s health during menopause: physical health, mental health, life satisfaction, and social involvement [18,19].

#### 2.5.3. Mood Status

The participants’ mood status was evaluated at Weeks 0 and 8 by the Profile of Moods Status second edition (POMS2)-brief [20,21,22]. Yokoyama et al. confirmed the reliability and validity of the POMS-brief translated into Japanese [22].

### 2.6. Outcome Measures

The participants’ scores for fatigue and mood conditions were the study’s primary outcomes; no secondary outcomes were set.

### 2.7. Statistical Analyses

Continuous variables with normality are presented as the mean  ±  standard deviation. The other data are presented as the median and interquartile (25th–75th). The amount of change in each score between the pre-intake test and the post-intake test is shown as Δ values. We used the Shapiro–Wilk test to evaluate the normality of data, parametric analyses for the normal-distribution data, and nonparametric analyses for the data that exhibited a non-normal distribution. For items that were clearly non-normally distributed, normality was not checked. The participants’ baseline characteristics were compared by a *t*-test or Wilcoxon’s two-sample test. The proportion of menopausal to non-menopausal participants was evaluated by Fisher’s exact test. Between-group comparisons for all efficacy endpoints after the treatment were performed using Wilcoxon’s two-sample test. To compare the changes from baseline to 4 or 8 weeks, we used Wilcoxon’s one-sample test. For multiple time points, Bonferroni-corrected Wilcoxon’s one-sample test was used. All statistical analyses were performed with Microsoft Excel 2019 and SPSS 26.0 (IBM, Armonk, NY, USA). Differences with *p*-values < 0.05 were considered significant.

## 3. Results

### 3.1. The Study Enrollment and the Participants’ Baseline Characteristics

**Figure 1:**
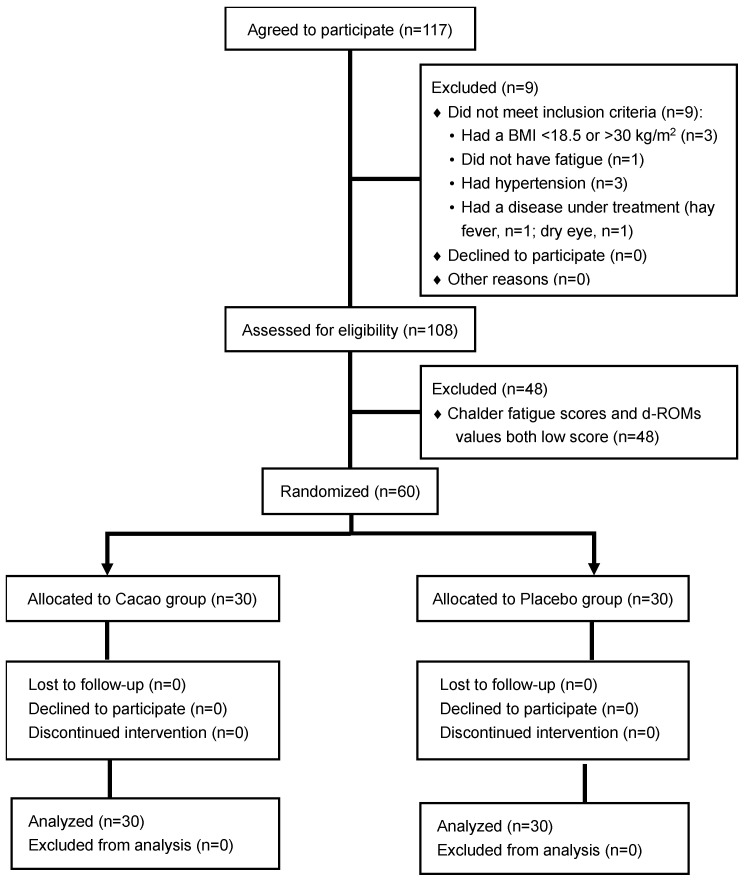
The flow of participant enrollment.

Figure 1 depicts the flowchart of the study enrollment. All 60 of the enrolled participants completed the study and were included in the efficacy and safety analyses. The treatment adherence was 100% with the exception of one case (98.2%) in the placebo group. The participant groups’ baseline characteristics are summarized in Table 1. No significant differences were identified between the cacao and placebo groups at baseline.

### 3.2. Efficacy Results

Table 2 provides the participants’ CFS values; the scores at both 4 and 8 weeks were significantly lower than the baseline scores in both groups. No significant difference was detected between the cacao and placebo groups’ CFS scores at any time point.

The POMS2 results are given in Table 3. Compared to the baseline values, after the 8-week intervention, both groups’ scores on all of the indices of negative mood were significantly decreased and both groups’ scores for all positive mood state indices were significantly increased. At baseline, there were no significant differences in any of the indices between the two groups, but after 8 weeks of treatment, the indices of negative mood (i.e., Depression-Dejection [DD], Fatigue-Inertia [FI], and Tension-Anxiety [TA]) were significantly lower in the cacao group than in the placebo group. Anger-Hostility (AH), another index of negative mood, showed a trend toward lower values in the cacao group compared to the placebo group. The overall evaluation result at 8 weeks, i.e., the Total Mood Disturbance (TMD), was significantly lower in the cacao group than in the placebo group. The change (Δ) in Vigor-Activity (VA, which was used as the index of positive mood) from baseline to 8 weeks of treatment was significantly higher in the cacao group versus the placebo group. The scores for DD, FI, and TA and the ΔVA values all improved with the intake of the beverage containing cacao flavanols.

Table 4 provides the data of the participants’ autonomic nervous system activity. There were no significant differences at any time points between or within the cacao and placebo groups. After 8 weeks of treatment, the LF component showed a trend toward lower values in the cacao group compared to the placebo group.

### 3.3. Safety Results

No serious adverse events were identified in this study. Seven adverse events occurred in seven of the placebo-group participants: rib pain (*n* = 1), shoulder pain (*n* = 1), neck pain (*n* = 1), arm pain (*n* = 2), fever (*n* = 2). In the cacao group, 10 adverse events occurred in nine subjects: headache (*n* = 1), toothache (*n* = 1), common cold symptoms (*n* = 1), fever (*n* = 1), acute sinusitis (*n* = 1), orthostatic dysregulation (*n* = 1), subconjunctival hemorrhage (*n* = 1), constipation (*n* = 1), and diarrhea (*n* = 2). There were no significant differences in the occurrence of adverse events between the groups. All identified adverse events were considered to have a clear cause, were considered to be transient or accidental, and were therefore deemed to be unrelated to the intervention, with no side effects observed.

## 4. Discussion

We conducted a randomized, double-blind, placebo-controlled pilot study to evaluate the effects of flavanol-rich cacao extract on fatigue and mood in healthy middle-aged Japanese women, and the results demonstrated that the intake of cacao flavanols improved the participants’ various mood conditions. In addition, no side effects from the intervention were observed throughout the study, suggesting that cacao flavanols are well tolerated by healthy middle-aged women.

The participants’ CFS scores at both 4 and 8 weeks were significantly lower than the baseline scores in both groups (*p* < 0.001). Sathyapalan et al. reported that an 8-week daily consumption of high-cacao polyphenol chocolate (160 mg of catechins and procyanidins) had a beneficial effect in improving the fatigue of subjects with chronic fatigue syndrome [5]. Our present study’s pre- and post-intervention comparisons revealed results that are similar to those of Sathyapalan et al., but we observed no significant difference in the CFS scores between the cacao and placebo groups. The difference between the results of the two studies may have occurred because the CFS is a questionnaire developed for chronic fatigue syndrome [17], and it may thus not have been appropriate for use in a population of healthy middle-aged women. Our participants were relatively healthy and did not have very high scores of fatigue at the baseline (Table 2). In addition, the level of polyphenols (catechins and procyanidins) in our study’s cacao beverage was 120 mg, which is lower than that of the chocolate in the Sathyapalan study.

Our present POMS2 results suggest that in middle-aged women, the consumption of flavanol-rich cacao extract reduces negative mood features, such as depression, fatigue, and anxiety, and improves the positive mood features vigor and activity. Gracia-Yu et al. reported that an additional daily consumption of 10 g of cocoa-rich chocolate by postmenopausal women could have a slight impact on their perception of the state of their health, although those authors noted that this was without modifications of the women’s health-related QoL or the dimensions that compose it [23]. One of our earlier studies demonstrated that in both middle-aged women and middle-aged men, a daily consumption of dark chocolate increased QoL scores [24]. Improving QoL and improving vigor are similar and the difference is ambiguous, and our present result regarding vigor can be understood in the same context. Other studies have used cocoa-rich or dark chocolate as a test food, whereas we used a beverage containing cacao flavanols (not chocolate). Taken together, the past and present findings suggest that the improved mood observed in the present study’s middle-aged women was due to cacao flavanols.

An improvement of mood state by flavanol-rich cacao extract may be mediated by antioxidant effects. During reproductive aging, oxidative stress in the form of free radicals and antioxidant deficiencies has been directly linked to the decline of estrogen [25]. The depressive symptom score has shown to be independently associated with a high 8-hydroxydeoxyguanisine level in middle-aged women, suggesting a link between mood disorder and oxidative stress [26]. Flavonoids have also been reported to protect cells such as neurons from oxidative stress [27]. The underlying mechanisms require further investigation.

The present study’s cacao group showed a decreasing trend in the LF component of autonomic nervous system activity after 8 weeks of cacao flavanol intake compared to the placebo group, although the difference was not significant. This result may indicate that the sympathetic nervous system was relieved by cacao flavanols. Duarte et al. reported that a single dose of dark chocolate increased both parasympathetic modulation and heart rate variability in healthy subjects [28]. One possible reason why our results differ from theirs is that the test food we used was cacao flavanols rather than dark chocolate. In addition, environmental factors, circadian rhythms, respiration, the menstrual cycle, and mental states are known to influence heart rate variability [29,30]. The difficulty in matching these variables for the participants in our present investigation may have meant that their autonomic activity levels were not properly measured and that the effect of cacao flavanols could not be verified.

The cacao flavanol beverages used in this study contain small amounts of methylxanthines, including caffeine and theobromine. Methylxanthines were reported to have psycho-stimulant effects [31,32]. Smit et al. reported that 19 mg of caffeine and 250 mg of theobromine were effective on both a mood state (“energetic arousal”) and some cognitive functions in adult males and females [32]. However, the theobromine dose used in the present study was lower than that in the Smit et al. study. In addition, another study by Smit et al. found no significant effects on mood state at doses similar to those used herein (8 mg of caffeine and 100 mg of theobromine) [32]. The studies by Smit et al. also differ from our present investigation in that they evaluated the more immediate effects (2 h) after a single intake. In addition, a previous study reported that 250 mg of cocoa flavanols, about the same amount as in this study, improved mental fatigue and some cognitive function in young healthy adults [33]. The possibility that the methylxanthines that we used in this study may be partially responsible for the observed mood state thus cannot be ruled out, but we suspect that primarily the cacao flavanols contributed to this effect.

Our study has some limitations. Anti-oxidative effects were not evaluated, and the relationship between the improvement of moods and the antioxidant action of cacao flavanols cannot be directly explained. Moreover, we did not conduct a daily dietary survey of the participants. The lack of information on daily energy intake and macronutrient composition limits the evaluation of whether these factors influenced the effects of the cacao flavanols. Analyzing the dietary information of the participants in future studies will give more accurate results. The generalizability of the findings obtained in this study is limited by the fact that it was conducted on Japanese women. Studies with subjects from other regions and ethnicities are desirable. Despite these limitations, the strength of this study is that it was a double-blind, placebo-controlled trial, which is not a conventional approach to examine the efficacy of food ingredients on health.

## 5. Conclusions

Compared to the intake of a placebo beverage, the consumption of a beverage containing flavanol-rich cacao extract significantly improved the participants’ negative mood and promoted positive mood, suggesting that cacao flavanols may be a useful food material that can improve middle-aged women’s mood condition and support their active lives.

## Figures and Tables

**Table 1:** The participants’ background characteristics.

	Placebo *n* = 30	Cacao *n* = 30	*p*-Value
Age, years	51.6 ± 3.6	52.4 ± 3.4	0.3996 ^a^
Body composition:			
Height, cm	157.9 ± 5.3	158.0 ± 4.0	0.9192 ^a^
Weight, kg	56.4 ± 7.6	55.2 ± 6.5	0.5347 ^a^
BMI, kg/m^2^	22.6 ± 2.4	22.1 ± 2.3	0.4375 ^b^
Cardiovascular parameters:			
Systolic blood pressure, mmHg	124.0 ± 14.5	121.5 ± 14.3	0.5035 ^a^
Diastolic blood pressure, mmHg	76.3 ± 11.1	75.8 ± 12.8	0.8721 ^a^
Heart rate, bpm	73.8 ± 8.9	69.6 ± 8.5	0.0686 ^b^
Menopausal status:			
Menopausal/not menopausal, *n*	12/18	12/18	0.4379 ^c^
Oxidative stress markers:			
d-ROMs, U.CARR	396.5 ± 51.1	398.5 ± 46.1	0.8700 ^a^
BAP, μM	2402.1 ± 146.6	2426.5 ± 182.9	0.5713 ^a^
Chalder Fatigue Scale score	28.0 (24.0–31.8)	26.5 (24.0–28.8)	0.5237
MHR-QOL:			
Physical symptom score	19.0 (17.0–23.0)	19.5 (16.0–22.0)	0.8820
Psychological symptom score	25.5 (17.3–28.0)	25.0 (23.0–28.0)	0.8011

Values are mean ± SD or median and interquartile ranges (25th–75th). Participants in menopause were defined as those who answered “no menstruation” in the questionnaire. ^a^ Student’s *t*-test or Welch’s *t*-test. ^b^ Wilcoxon two-sample test. ^c^ Fisher’s exact test. d-ROMs: derivatives-reactive oxygen metabolites, BAP: Biological Antioxidant Potential, MHR-QOL: Menopausal Health-Related Quality of Life.

**Table 2:** The participants’ Chalder Fatigue Scale (CFS) score.

	Placebo *n* = 30		Cacao *n* = 30		*p* vs. Placebo	*p* vs. Week 0	
						Placebo	Cacao
Week 0	21.5	(18.0–26.5)	22.0	(16.3–25.8)	0.9823	–	–
Week 4	15.0	(13.0–19.8)	14.5	(13.3–20.0)	0.8643	**0.0000**	**0.0002**
Δ	−5.0	(−13.0 to −2.0)	−5.5	(−9.0 to −2.0)	0.4910	–	–
Week 8	13.0	(9.3–20.0)	14.0	(10.3–16.8)	0.5991	**0.0001**	**0.0000**
Δ	−9.0	(−12.8 to −1.5)	−8.5	(−12.0 to −5.5)	0.8067	–	–

Values are median and interquartile ranges (25th–75th). Δ: the change from Week 0. *p*-values less than 0.05 are highlighted in bold.

**Table 3:** The participants’ Profile of Moods Status second edition (POMS2) scores.

		Placebo *n* = 30		Cacao *n* = 30		*p* vs. Placebo	*p* vs. Week 0	
							Placebo	Cacao
AH	Week 0	51.0	(45.5–65.5)	50.0	(40.0–52.0)	0.0622	–	–
	Week 8	47.0	(43.5–52.8) *	43.0	(40.0–50.0)	0.0917	**0.0046**	0.0789
	Δ	−5.0	(−12.0 to 1.5)	−1.0	(−7.0 to 0.0)	0.2439	–	–
CB	Week 0	52.0	(46.0–60.5)	50.0	(46.0–58.5)	0.4853	–	–
	Week 8	46.0	(40.8–50.5) *	41.5	(40.0–50.5) *	0.1683	**0.0004**	**0.0007**
	Δ	−3.0	(−15.3 to 0.0)	−5.0	(−9.0 to 0.0)	0.8938	–	–
DD	Week 0	50.0	(43.0–57.3)	46.5	(41.0–54.0)	0.2520	–	–
	Week 8	46.0	(42.3–52.5) *	43.0	(41.0–46.8) ^#,^*	**0.0459**	**0.0135**	**0.0017**
	Δ	−2.0	(−9.0 to 0.0)	−1.0	(−8.5 to 0.0)	0.7343	–	–
FI	Week 0	54.0	(47.0–60.0)	50.5	(45.0–57.5)	0.2658	–	–
	Week 8	45.0	(43.0–49.0) *	42.0	(37.0–46.0) ^#,^*	**0.0108**	**0.0001**	**0.0000**
	Δ	−6.0	(−9.8 to −0.5)	−6.0	(−10.8 to −2.0)	0.8067	–	–
TA	Week 0	52.0	(44.8 to 61.0)	48.0	(42.3 to 54.0)	0.0719	–	–
	Week 8	49.0	(42.0–52.0) *	44.0	(39.5–48.8) ^#,^*	**0.0184**	**0.0042**	**0.0006**
	Δ	−5.0	(−9.8 to 1.5)	−5.0	(−7.0 to 0.0)	0.7777	–	–
VA	Week 0	43.5	(38.5–47.8)	44.0	(38.0–50.0)	0.7895	–	–
	Week 8	46.0	(43.0–54.0) *	50.5	(45.0–57.0) *	0.2534	**0.0104**	**0.0000**
	Δ	2.5	(0.0–8.5)	7.0	(2.3–11.8) ^#^	**0.0422**		
F	Week 0	44.0	(41.0–49.0)	46.0	(38.5–57.5)	0.3280	–	–
	Week 8	49.0	(44.3–52.8) *	51.0	(46.0–58.0) *	0.2950	**0.0015**	**0.0393**
	Δ	4.0	(0.0–8.0)	3.0	(0.0–8.0)	0.4704	–	–
TMD	Week 0	52.5	(47.5–63.0)	51.0	(45.5–54.8)	0.1167	–	–
	Week 8	47.5	(42.3–50.0) *	42.0	(39.0–47.0) ^#,^*	**0.0141**	**0.0001**	**0.0000**
	Δ	−4.5	(−9.8 to −0.3)	−6.0	(−9.8 to −2.0)	0.4723	–	–

Values are median and interquartile ranges (25th–75th). # *p* < 0.05 vs. placebo. * *p* < 0.05 vs. Week 0. *p*-values less than 0.05 are highlighted in bold. Δ: the change from Week 0, AH: Anger-Hostility, CB: Confusion-Bewilderment, DD: Depression-Dejection, FI: Fatigue-Inertia, TA: Tension-Anxiety, VA: Vigor-Activity, F: Friendliness, TMD: Total Mood Disturbance.

**Table 4:** The participants’ autonomic nervous system activity.

		Placebo *n* = 30		Cacao *n* = 30		*p* vs. Placebo	*p* vs. Week 0	
							Placebo	Cacao
HF	Week 0	300.12	(160.96–439.75)	373.89	(141.85–836.94)	0.3593	–	–
	Week 8	289.65	(151.15–650.56)	242.59	(136.31–551.88)	0.6152	0.6583	0.1064
	Δ	−33.21	(−168.88 to 248.69)	−41.83	(−360.44 to 79.78)	0.1474	–	–
LF	Week 0	312.39	(110.23–482.11)	171.05	(102.18–345.92)	0.3077	–	–
	Week 8	192.17	(114.64–394.50)	144.58	(84.45–198.98)	0.0546	0.8936	0.2536
	Δ	−4.13	(−112.38 to 154.64)	−15.43	(−147.02 to 56.96)	0.4420	–	–
Sympathetic index	Week 0	2.33	(1.65–4.55)	1.67	(0.86–4.24)	0.0891	–	–
	Week 8	2.06	(0.98–3.63)	1.53	(0.83–3.57)	0.3912	0.7036	0.5857
	Δ	−0.11	(−2.18 to 1.56)	0.20	(−0.88 to 0.54)	0.4871	–	–
Parasympathetic index	Week 0	0.30	(0.18–0.38)	0.37	(0.19–0.54)	0.0891	–	–
	Week 8	0.33	(0.22–0.51)	0.40	(0.22–0.55)	0.3912	0.4048	0.5304
	Δ	0.02	(−0.11 to 0.19)	−0.03	(−0.11 to 0.08)	0.3750	–	–
LF/HF ratio	Week 0	0.82	(0.39–1.48)	0.43	(0.28–1.08)	0.0850	–	–
	Week 8	0.77	(0.35–1.26)	0.45	(0.24–0.88)	0.1297	0.9918	0.3764
	Δ	−0.00	(−0.25 to 0.52)	−0.08	(−0.17 to 0.14)	0.5844	–	–

Values are median and interquartile ranges (25th–75th). Δ: the change from Week 0. HF: high-frequency, LF: low-frequency.

## Data Availability

The study data are available upon reasonable request to the corresponding author.
